# Letrozole administration effects on the P450aromatase expression and the reproductive parameters in male sheep (*Ovis aries*)

**DOI:** 10.1590/1984-3143-AR2023-0099

**Published:** 2023-12-01

**Authors:** Antônio Francisco da Silva Lisboa, Túlio Teruo Yoshinaga, Antonio de Sousa, Marcílio Nichi, Alejandro Esteller-Vico, Antônio Chaves de Assis

**Affiliations:** 1 Departamento de Cirurgia, Faculdade de Medicina Veterinária e Zootecnia, Universidade de São Paulo, São Paulo, SP, Brasil; 2 Colégio Técnico de Teresina, Universidade Federal do Piauí, Teresina, PI, Brasil; 3 Departamento de Reprodução Animal, Faculdade de Medicina Veterinária e Zootecnia, Universidade de São Paulo, São Paulo, SP, Brasil; 4 Department of Biomedical and Diagnostic Science, College of Veterinary Medicine, University of Tennessee, Knoxville, TN, USA

**Keywords:** steroidogenesis, estradiol, testosterone, aromatase inhibitors, male reproduction

## Abstract

Letrozole comprises a non-steroid aromatase inhibitor that has been applied as a preventive for many uses, such as breast cancer prevention, treatment of hormonal dysfunction, and male infertility. Precisely in Northeast Brazil, ovine consist of the leading livestock produced, and their reproduction in captivity has been demonstrated difficult. Thus, we hypothesized whether the application of letrozole will improve male sheep reproduction. One group of 6 animals received a daily dosage of 0.5mg/Kg of letrozole for 60 days, while the other six animals were used as control. Samples were collected from control and treated animals after 30 and 60 days of the experiment. Blood samples were collected to measure the steroid hormone levels. Semen was collected from control and treated groups using an artificial vagina and cryopreserved for spermatozoa morphology and CASA analysis. The testicles were collected for histological analysis, gene expression, and immunohistochemistry of P450aromatase protein. Hormone levels demonstrate no differences in the estradiol/testosterone levels among the control and both treated groups. Immunohistochemistry analysis revealed the presence of P450aromatase protein in spermatogonia cells and Leydig cells in the control and treated groups in both periods analyzed. Moreover, there were no differences in the P450aromase gene expression in the control and treated group. Morphological analysis of the spermatozoa revealed that letrozole treatment did not affect mitochondrial activity or cause any deformities. In addition, motility parameters in the sperm from the treated group were not affected by letrozole treatment compared to the control group. Morphological analysis of the testis demonstrated that letrozole treatment increase the seminiferous tubule area but no signs of germ cell damage. Our results show that letrozole has a morphological effect on the testicles in the ovine model but no pathological or severe effect caused by hormone level imbalance. Overall, letrozole comprises a non-steroid aromatase inhibitor, which can be applied to ovine reproduction.

## Introduction

The female and male germline differentiate into gametes through specific processes called oogenesis and spermatogenesis, respectively (Larose et al., 2019). In both processes, the sex steroid hormones such as estradiol and testosterone play essential roles in gamete maintenance and differentiation (Morohashi et al., 2013). During gametogenesis, sex steroid hormone synthesis occurs in the gonads in response to the follicle stimulant hormone (FSH) and luteinizing hormone (LH) either in the ovaries and testes (Yaron and Levavi-Sivan, 2011). Although the Sertoli cells play a central role in testicular spermatogenesis, protecting, nourishing, and supporting germ cells (Ni et al., 2019), the Leydig cells are the primary source of testosterone synthesis (Zirkin and Papadopoulos, 2018). Testosterone was considered for many years as the sole steroid hormone responsible for spermatogenesis. However, some studies demonstrated that estrogens also play an essential role in this process (Carreau et al., 2012; Carreau and Hess, 2010). Testosterone and estradiol are structurally similar molecules, whereby the testosterone is converted into estradiol by cytochrome aromatase *cyp19* P450 enzyme (Cornil, 2018).

P450 cytochrome aromatase (P450arom) comprises an enzymatic complex that catalyzes androgens in estrogens not only in the gonads but also in several tissues such as the brain, breast, adipose tissue, skin, placental cells, and bone (Carreau et al., 2007; Conley and Hinshelwood, 2001; Czajka-Oraniec and Simpson, 2010). In adult gonads, P450arom are usually found in the Leydig and granulosa cells in the testis and ovary, respectively (Baufeld and Vanselow, 2018; Zirkin and Papadopoulos, 2018). Furthermore, the application of aromatase inhibitors was usually prescribed for young and adult men who presented health problems related to steroidal hormone levels (Ronde and Jong, 2011), and also to treat cases of male infertility (Schlegel, 2012). Letrozole has been used to treat male infertility for years, which has different causes, such as autoimmune cases and steroid hormone deficiency (Schlegel, 2012). Due to the aromatase inhibitor effect, letrozole prevents estradiol synthesis, increasing testosterone levels and improving sperm concentration (Shuling et al., 2019). Besides the reproduction functions, there are other roles of aromatase and steroid hormones in different tissues (Cornil, 2018; Rosenfeld et al., 2018; Ulhaq, 2019), and more recently, aromatase has been associated with breast cancer development (Zhao et al., 2016). In this regard, aromatase inhibitors were also employed as treatment or in order to prevent cancer development in women (Pistelli et al., 2018).

Although aromatase inhibitors present an excellent perspective in breast cancer therapy, there are side effects of their use in reproduction (Dent et al., 2011). In testis, aromatase inhibition and, hence, estrogen deficiency demonstrated a direct influence on the morphology of seminiferous tubules and interstitial tissue in mice (Kondarewicz et al., 2011). The imbalance of steroidal hormones can cause testis tissue abnormalities in prolonged treatment of letrozole (Arroyo et al., 2021; Pilutin et al., 2014). The increase in the estradiol levels can promote Leydig cell hypertrophy and hyperplasia and increase inflammatory cell numbers in transgenic mice expressing high aromatase levels (Li et al., 2006). In addition, estrogen produced by P450 aromatase activity is related to controlling several reproductive mechanisms in germ cells from different species (Rosati et al., 2016, 2019, 2020).

Despite the effects of aromatase inhibitors, the long-term treatment and the effects of letrozole on ovine reproductive performance under *in vivo* conditions have not yet been reported in the literature. Thus, the present study aimed to evaluate the effect of letrozole, a non-steroid aromatase inhibitor, on the reproductive parameters in adult male sheep testis reared under intensive management conditions in Northeast Brazil. In this regard, we hypothesized whether letrozole would affect on ovine male fertility. In order to evaluate these effects, the hormonal levels were measured, as well as P450aromatase expression. Specific analyses were conducted on the spermatozoa morphology and motility parameters.

## Materials and methods

### Animals rearing conditions and ethical statements

Twelve adult Dorper rams aged between 12 and 14 months (total body weight ±30 Kg) were reared in a confinement system (4º30'57” S, 42º37'30” W) located in the State of Piaui, Brazil. The animals were maintained under a natural photoperiod and fed twice a day with corn silage. All procedures followed the guidelines for the care and use of laboratory animals of the School of Veterinary Medicine and Animal Science of the University of São Paulo, certificate CEUA/FMVZ #7512260617.

### Letrozole treatment

A pilot test established a proper dosage prior the experimental trial. Three different dosages were prepared, 0.1, 0.5, and 1 mg/Kg of letrozole, and administrated orally in three groups with three animals. Only one dosage was administered, and blood samples were collected before and after letrozole administration to measure serum levels of estradiol and testosterone. Details about the blood collection and the hormone analysis will be in the following sections.

After the pilot test, the dosage of 0.5 mg/Kg was established for the experimental trial. One-year-old ram animals were randomly divided into four groups: 30-day control (G1), 60-day control (G2), 30-day treatment (G3), and 60-day treatment (G4). The animals in the control groups (i.e., G1 and G2) were administered a placebo orally during the experiment. In contrast, the animals in G3 and G4 were orally administered 0.5 mg/kg letrozole daily during the respective periods. After 30 days of treatment, reproductive parameters were collected from G1 and G3 groups, and G2 and G4 were collected after 60 days of treatment. Also, testis samples from all four groups were collected for histology and gene expression analysis.

### Free estradiol and testosterone circulating radioimmunoassay in the blood

Blood samples were collected in vacuum tubes by jugular vein puncture after 30 and 60 days of treatment. Four mL of blood were centrifuged at 3000 x *g* for 10 minutes. The serum was carefully removed from the upper layer, transferred to a new 1,5 mL tube, and stored until analysis. Testosterone and estradiol levels were measured by radioimmunoassay on serum samples using chemiluminescence (Immulite 1000, Siemens Healthcare Diagnostics, Tarrytown, NY). Estradiol levels (Immulite 1000, PIL1KE2D) were measured as previously described (Habeeb et al., 2018). The limit of detection was 15 pg/mL, and precision for low control was 9.9%, medium control 7.8%, and high control 4.3%. Testosterone level (Immulite 1000, L1KTW2) was measured as previously described (Van Emon et al. 2013). The detection limit was 20 pg/mL, and precision for low control was 16.3%, medium control 11.7% and high control 8.3%. All samples were run in triplicates according to the manufacturer’s instructions and performed on the same day.

### Reproductive parameters and semen collection

The testicle circumferences were measured in all animals, and ram semen was collected using an artificial vagina. Then, the semen was diluted 1:1 with an extender solution containing 0.25 M TRIS, 0.6 M D-Fructose, 20% of egg yolk, and 10% of glycerol in distilled water. The diluted semen was pipetted into 0.5 mL straws placed in the TK 3000 CSE® frozen machine (TK Tecnologia, Brazil) to cool down the straws and transferred to liquid nitrogen until further analysis.

### Spermatozoa morphology

Acrosome integrity was measured using a Fast-Greed/Rose-bengal solution (Pope et al., 1991). The staining solution consists of a 1% Fast green FCF, 1% Rose-bengal in 40% ethanol in a 0.1 M citric acid, and 0.2 M disodium phosphate buffer. The procedure was performed as follows. A 5 µL aliquot of semen was mixed with 5 µL Pope stain in a centrifuge tube and incubated in a thermoblock at 37°C for 60 seconds. Then, semen samples were smeared in glass slides and mounted for microscopic analysis. At least 200 spermatozoa cells were counted in each sample and raked as intact acrosome or damaged acrosome. Intact acrosomes comprise the strong lilac-purple acrosome region stain with a darker stain in the pos-acrosomal region. In contrast, a damaged acrosome comprises a pink stain in the acrosome region, with a lighter stain in the pos-acrosome region (Nichi et al., 2006).

The spermatozoa plasmatic membrane integrity was also evaluated using an eosin-nigrosine stain as described by (Barth and Oko, 1989). Briefly, a 5 µL semen aliquot was mixed well with 100 µL of 1% aqueous eosin-y solution in a centrifuge tube and incubated for 15 seconds in agitation. Then, 100 µL of 10% aqueous nigrosine solution was added to the tube and well-mixed. Two drops of the mixture were thinned and smeared in glass slides and let air dry. After the dry step, the slides were mounted with properly mounting media with cover glass. The spermatozoa were analyzed by the eosin-nigrosine stain pattern. Spermatozoa with intact plasmatic membranes will present a white or translucent appearance because the eosin will not stain them and will contrast with the dark nigrosine background. In contrast, spermatozoa with damaged membranes will be stained by eosin. At least 200 spermatozoa were counted in each slide and ranked as intact membrane (not stained) and damaged membrane (stained), respectively.

### Computer Assisted Sperm Analysis (CASA) and sperm mitochondrial activity

The semen samples were submitted to computer analysis of sperm motility (CASA) using IVOS II System v. 12.3 (Hamilton-Thorne, USA). CASA analysis was adapted from Crespilho et al. (2017). Frozen semen was thawed and warmed to 38°C. Then, 6 µL of semen was pipetted into the specific machine 20 μm-deep 4-chamber. A minimum of 1000 cells were analyzed in at least eight randomly selected fields, with 30 frames acquired per field at a frame rate of 60 Hz. The following parameters were measured: curvilinear velocity (VCL), average path velocity (VAP), velocity in a straight line (VSL), lateral amplitude movement (ALH), beating cross frequency (BCF), Straightness (STR), and linearity (LIN).

Also, semen samples were submitted to the Diaminobenzidine test (DAB) following (Hrudka, 1987). Briefly, 25 mL of semen was incubated with 1mg/mL DAB solution in 0.1M PBS for 1 hour. After incubation, the semen was smeared in glass slides and fixed using Phosphate buffered formalin 10% for 10 minutes. The slides were rinsed in 0.1M PBS and let dry in an air chamber protected from light. Mitochondrial cytochemistry was evaluated in a Phase-contrast microscopy. At least 200 spermatozoa were counted in each slide and ranked according to the color intensity from the spermatozoa middle piece. Spermatozoa ranked “I” middle piece full colored indicate high mitochondrial activity. “II” more than half of the middle piece indicated mean-high activity. The “III” middle piece with less than half of the colored segments lower mitochondrial activity and the “IV” middle piece without stain indicates the absence of mitochondrial activity.

### Spermatozoa oxidative stress resistance assay

The spermatozoa were analyzed by oxidative stress resistance assay as described previously (Ohkawa et al., 1979). Briefly, 200µL of fresh thawed semen were incubated with 50 µL of ascorbic acid solution (20 mM in 0.1 M PBS) and 50 µL of iron sulfate solution (4 mM in 0.1M PBS) for 90 minutes in water bath to induce lipidic peroxidase activity. Then, 600 µL of 10% trichloride acetic acid water solution (w/V) was centrifuged at 20000 x g for 15 minutes at 5°C to precipitate the proteins. The 700 µL from the supernatant was transferred to a 4 mL cryotube, and then 700 µL of 1% Tio barbituric acid was added to the tube and incubated at 100°C for 15 minutes. After the incubation, the tubes were immediately transferred to water at 5°C to stop the reaction. The solution was read in the spectrophotometer at 532 nm. The spermatozoa Tio barbituric resistance (TBARS) is calculated by the ratio of the Marlon curve index MDA = (58.838 + 19272 x absorbance) x 10^6^ by the spermatozoa concentration multiplied by 10^6^

### Histological analysis

At the end of each period, the groups had their testicles removed and fixed in Bouin's solution at 4 °C for 24 hours, embedded in paraffin, and sliced into 5-µm thickness sections. The sections were then stained with hematoxylin and eosin (HE) for morphological observation of testicular tissue.

Testis sections were also subjected to electronic microscope analysis. Testis samples were fixed in Karnovisk solution. Then, the samples were dehydrated in ethanol series and embedded in histological resin. 60nm ultra-thin slices were obtained in copper mesh and counterstained with 2% uranyl acetate and 0.5% lead citrate in distilled water for 5 and 10 minutes, respectively. Then, images were obtained in a transmission electron microscope.

The immunohistochemical (IHC) procedure was performed as follows. Testis sections were deparaffined, re-hydrated, submitted to antigen retrieval process using citrate buffer (pH 6.0), and heated in an electric pressure cooker for 5 min. After cooling at room temperature for 20 min, the sections were rinsed twice in 0.1M PBS for 5 min, followed by the blocking of endogenous peroxidase using Peroxidase Block for 30 min using 3% Hydrogen Peroxide in Methanol. The sections were rinsed in 0.1M PBS for 5 min, and the protein block was performed using Protein Block for 30 min using TNB blocking buffer (Perkinelmer, USA). Incubation was performed with a primary anti-aromatase antibody (Anti-rabbit/mouse (1/400- ab18995, Abcam, Cambridge, MA, USA) validated by other studies (M.A.M. Arroyo et al., 2016; Santos et al., 2014; Santos et al., 2017) in 1:200 dilution in PBS, in a humid chamber for 16 h. The sections were rinsed in 0.1M PBS for 5 min and incubated with secondary antibody EnVision™+ Dual Link System-HRP secondary antibody (Dako Ominis, Agilent, USA) for 30 min. Samples were washed in PBS for 5 min and incubated in a DAB chromogen. The slides were washed in distilled water, counterstained with hematoxylin, and mounted. The intensity of the immunostaining was evaluated through a subjective method by observation in an optical microscope (Ribeiro et al., 2013). Samples were photo-documented using an Olympus® BX61VS microscope.

### RNA extraction, cDNA synthesis, and qRT-PCR analysis

Sheep testis samples were fixed in RNA *Later* (Invitrogen) solution for 12 h and frozen at -80 °C for further processing. The samples were homogenized using L-Beader cell disruptor 6 (Loccus), and the total RNA was extracted with *TRIzol^TM^ Reagent* (Invitrogen). The RNA samples were quantified in a Biodrop *µ*LITE. For each sample, 1 µg of RNA was purified with TURBO™ DNase (Invitrogen), and retrotranscription was performed using a *High-Capacity cDNA Reverse Transcription Kit* (Applied Biosystems). All procedures followed the manufacturer's instructions. The expression analysis was performed using *Select SYBR Green Master Mix* (Applied Biosystems) in *Step One Plus Real-Time PCR System* (Applied Biosystems). The two-step qPCR condition was as follows: 40 cycles of 95 °C for 30 s and 60°C and the relative expression levels were measured by the 2^^-ΔΔCT^ method. Each sample was prepared in triplicate, together with the endogenous controls. GAPDH was used as an endogenous control. The Cytochrome P450 aromatase-specific *primers* specific to the (Cyp19) enzyme and endogenous control genes are listed in Table 1.

**Table 1:** Primers sequences and amplicon size.

**Gene name**	**Primer sequence (5’ ← 3’)**	**Amplicon size** **in bp**	**Genbank accession number**
***cyp19***	Fw: TGAGGACCAGGTTGTCTCCT Rv: AAGTGGTCGTTGAGGGCAAT	126	NM_001123000.1
***actinb***	Fw: CGCAAGTACTCCGTGTGGAT Rv: TGTAACGCAGCTAACAGTCCG	148	NM_001009784.3

Primer sequence used for P450 aromatase gene expression. Beta-actin was used as endogenous control.

### Statistical analysis

The relative aromatase expression data were submitted to the Mann-Whitney non-parametric U test or one-way-ANOVA followed by Tukey’s post-test. The significant difference was attributed to p < 0.05. Statistical analyses were performed using the *GraphPad Prism* v. 9.1 software.

## Results

### Letrozole dose-hormone response

**Figure 1:**
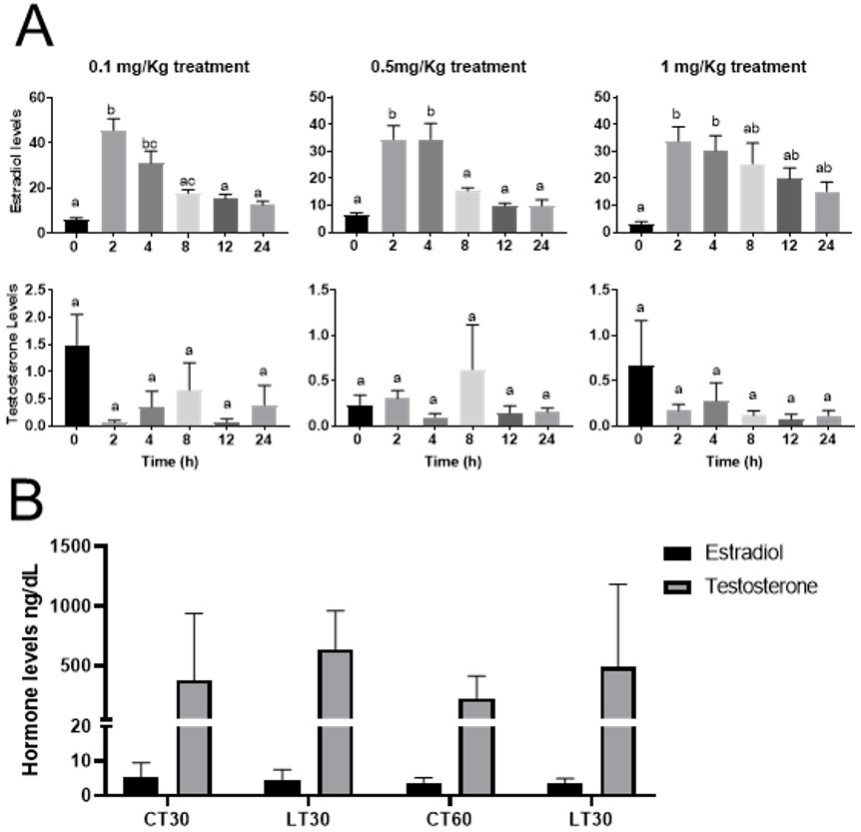
Pilot test of letrozole treatment. Hormonal levels of treatment animal during the first 24 hours (A). Hormonal levels of long-term letrozole treatment for 60 days (B). CT30 and CT60 are the control groups; LT30 and LT60 are treated animals for 30 and 60 days, respectively. Different letters represent significant differences for *p ≤* 0.05 One-way ANOVA analysis followed by Tukey’s test in A. Different letters represent significant differences for *p ≤* 0.05 Two-way ANOVA analysis followed by Tukey’s post-test in B. Data are presented as mean ± SD.

Firstly, we established a dose hormone response in a pilot trial. Treated animals demonstrated high estradiol levels 2 to 4 hours post-one letrozole administration in all concentrations tested (Figure 1A). After this period, estradiol levels decreased more prominent in the 0.1 and 0.5 mg/kg treatment, while 1 mg/kg treatment did not present a difference after 24 hours. Testosterone levels did not alter in any analyzed period (Figure 1A). After the pilot test, letrozole treatment were carried out daily with a dosage of 0.5 mg/Kg.

### Estradiol and testosterone levels on letrozole treated animals

Letrozole treatment animals presented ten times testosterone levels compared to estradiol after 30 and 60 days (Figure 1B). However, there is no difference in testosterone levels of between control and treated groups in both periods analyzed (Figure 1B).

### P450 aromatase immunolocalization and gene expression

**Figure 2:**
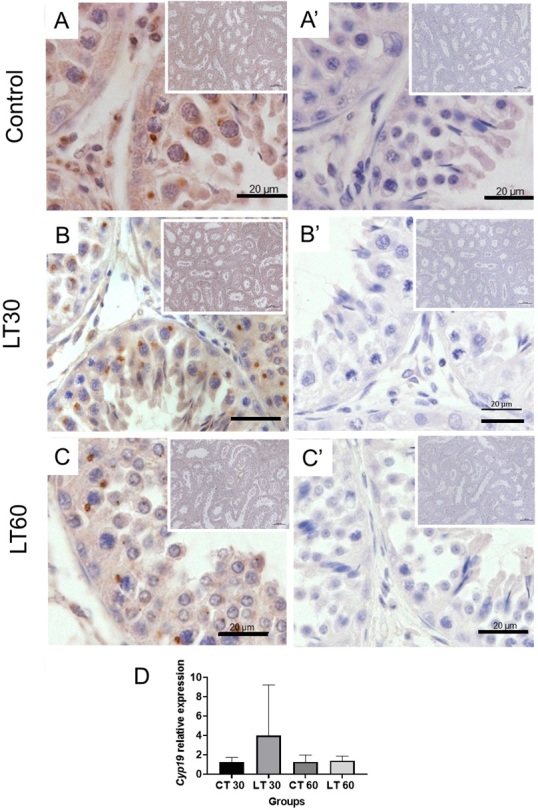
Expression analysis of P450 aromatase in testicles from animals treated with letrozole. Immunohistochemistry analysis Control (A); 30 days of Letrozole treatment, LT30 (B); 60 days of letrozole treatment LT60 (C). In the right column are the respective negative controls. Relative gene expression in control and treated animals (D). Scale bars indicate 20 μm.

P450 aromatase was immunolocalized in testes from treated animals. Positive immune reaction was observed mainly in Leydig cells, spermatogonial, and primary spermatocytes in all samples in control and treated groups (Figure 2A-C). Although positive maker in Leydig cells was prominent in all the cytoplasm, spermatogonia, and primary spermatocytes displayed an exciting pattern expression concentrated next to the nucleus, as indicated in Figure 2A-C and Table 2.

**Table 2:** Testicular cells P450 immunolocalization of letrozole treated animals.

**Cytochrome P450 aromatase**	**Control**	**30 days**	**60 days**
Leydig cells	+++	+++	+++
Sertoli cells	---	---	---
Myoid cells	---	---	---
Spermatogonia	+++	+++	+++
Spermatocyte	+++	+++	+++
Spermatids	----	---	---

Immunolocalization of the P450 aromatase enzyme in the testicle animals from the control, 30-day letrozole treatment, and 60-day letrozole treatment groups.

Gene expression analysis of P450 aromatase revealed no statistical differences. Nevertheless, the absolute number is at least three times higher in the 30-days treated group, while no differences were observed after 60 days of treatment, as shown in Figure 2D.

### Morphometric and histopathological analysis

**Figure 3:**
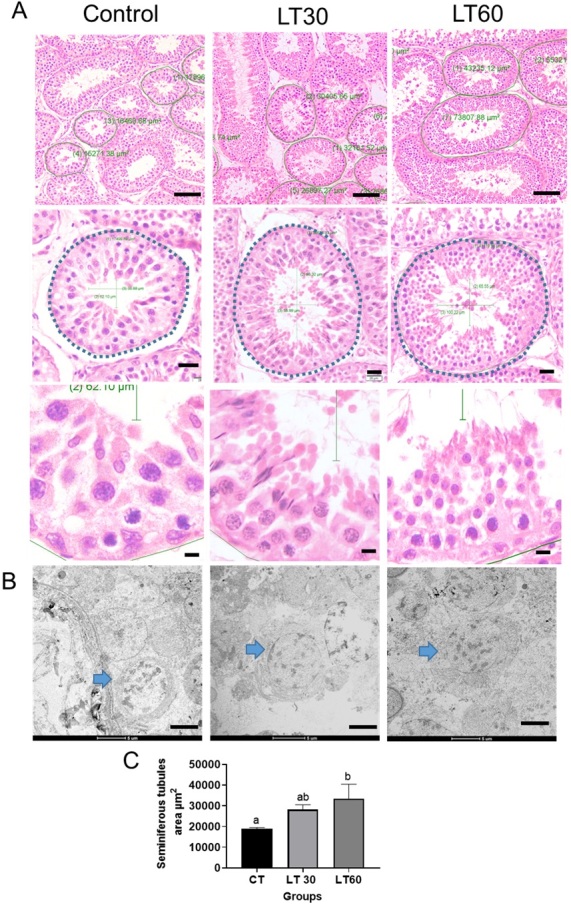
Histological analysis from testicles from letrozole-treated animals. Conventional histology showing seminiferous tubules from Control (CT) and letrozole-treated animals (LT30 and LT60) (A). Transmission electron microscopy showing spermatogonial cells (blue arrowhead). Seminiferous tubule area from control and letrozole-treated animals (B). Area measurement from seminiferous tubules (C). Different letters represent significant differences for *p ≤* 0.05 One-way ANOVA analysis followed by Tukey’s test. Data are presented as mean ± SD. Scale bars in A indicate 100 μm, 20 μm, 10 μm, and 5 μm in electron images from B.

The testes from letrozole-treated animals presented a high scrotal circumference compared to the control in both periods analyzed (Figure 3A). Ultrastructure histological analysis also revealed no pathological signs in the testis of treated animals compared to the control group. Normal tissue morphology was possible to observe as in the control group. (Figure 3B). In addition, histological analysis from testis samples revealed an increase in the seminiferous tubules total area and the lumen diameter in letrozole treated group in both periods analyzed compared to the control group (Figure 3C). No pathological signs or tissue damage were observed in the seminiferous tubules of treated animals.

### Spermatozoa analysis

The letrozole treatment did not affect spermatozoa morphology at any time (Table 3). The letrozole-treated animals did not present a statistical difference in spermatozoa concentration. Other analyses, such as plasma membrane integrity and acrosome integrity, also did not differ in both periods analyzed, as well as the oxidative stress parameter (Table 4). In addition, letrozole treatment did not affect the mitochondrial activity (Table 4) in the DAB assay and did not affect any parameters analyzed by CASA (Table 5).

**Table 3:** Morphological analysis of the spermatozoa of letrozole treated animals.

		**Time (days)**	
**Parameters**	**0**	**30**	**60**
**DEFMA**	4.25±2.71^a^	9.75±8.46^a^	9.0±8.02^a^
**DEFME**	2.5±2.17^a^	4.75±1.11^a^	2.66±1.76^a^
**DEFTOT**	6.75±4.81^a^	14.5±9.25^a^	11.66±9.66^a^

Morphological deformity analysis of the spermatozoa from letrozole treated animals **DEFMA** (%, Great defects), **DEFME** (%, minor defects) and **DEFTOT** (%, total defects).

**Table 4:** Acrosomal integrity and mitochondrial activity of the spermatozoa from letrozole treated animals.

	**Time (days)**		
**Parameter**	**0**	**30**	**60**
**CONC**	170000000±16832508.23^a^	225000000±40875828.23^a^	250000000±25658007.20^a^
**INTEGRO**	30.5±8.76^a^	31.0±1.35^a^	46.66±7.66^a^
**ACRO**	82.25±2.49^a^	83.5±1.19^a^	90.0±3.60^a^
**DAB I**	26.5±5.48^a^	34.5±7.02^a^	36.33±5.89^a^
**DAB II**	52.0±1.08^a^	23.5±6.58^b^	41.0±1.52^c^
**DABIII**	14.0±5.33^a^	6.25±1.18^a^	6.0±3.05^a^
**DAB IV**	7.75±3.11^b^	35.75±7.58^a^	16.66±4.84^b^
**TBARS**	1.45±0.15^a^	1.4±0.18^a^	3.08±1.55^a^

Spermatozoa parameters from animals treated with Letrozole. **CONC** (spermatozoa concentration), **INTEGRO** (plasma membrane integrity); **ACRO** (acrosome integrity); **DAB I** (High mitochondrial activity); **DAB II** (medium mitochondrial activity); **DAB III** (low mitochondrial activity); **DAB IV** (absence mitochondrial activity); **TBARS** (Oxidative stress susceptivity evaluation).

**Table 5:** CASA analysis of the spermatozoa from letrozole treated animals.

	**Time (days)**		
**Parameters**	**0**	**30**	**60**
**VAP**	77.37±9.66^a^	78.9±8.41^a^	65.66±1.79^a^
**VSL**	62.7±7.49^a^	63.52±5.75^a^	56.1±4.23^a^
**VCL**	126.17±20.39^a^	128.75±17.09^a^	97.0±4.36^a^
**ALH**	6.82±0.41^a^	6.57±0.26^a^	5.5±0.73^a^
**BCF**	28.6±2.53^a^	30.37±3.29^a^	20.7±1.61^a^
**STR**	78.75±2.53^a^	80.0±1.08^a^	85.0±4.04^a^
**LIN**	51.0±3.82^a^	52.0±2.04^a^	60.0±6.43^a^
**MOTILE**	34.0±8.74^a^	41.75±6.15^a^	42.0±13.27^a^
**PROGR**	13.0±5.81^a^	13.0±1.96^a^	10.33±1.45^a^
**RAPID**	16.75±6.75^a^	18.25±2.65^a^	14.0000±0^a^
**MEDIUM**	17.25±4.76^a^	23.25±5.85^a^	27.33±13.86^a^
**SLOW**	11.75±3.01^a^	14.25±2.71^a^	4.33±1.20^a^
**STATIC**	54.25±10.37^a^	44.00±7.59^a^	54.33±15.02^a^

CASA parameters adult ovine males after 30 and 60 days of letrozole treatment. **VAP** (Average path velocity); **VSL** (Rectilinear velocity); **VCL** (Curvilinear Velocity); **ALH** (head lateral movement amplitude); **BCF** (Cross beating frequency); **STR** (VSL/VAP, % rectilinearity) **LIN** (VSL/VCL, % linearity); **MOT** (% Motility); **PROG** (% progress motility); **RAPID** (% Quick speed spermatozoa); **MEDIUM** (% medium speed spermatozoa); **SLOW** (% lower speed spermatozoa); **STATIC** (% Static spermatozoa).

## Discussion

Several studies have reported Aromatase inhibitor administration in recent years (Akça et al., 2022). Letrozole, in a specific manner, has been used in cancer treatment, sex reversal studies, and treatment of male infertility (Nunes et al., 2021; Shen et al., 2015; Yang et al., 2021). In this study, we aimed to address the effects of letrozole administration in male ovine models for the first time. Letrozole treatment with 0.5mg/Kg dosage could increase the testis size and alter the estradiol/testosterone serum levels balance in the first hours of treatment. Moreover, the application of letrozole did not affect the quality of spermatozoa or cause any pathological effect on treated animals.

Steroid hormones play essential roles in reproduction; estradiol and testosterone are primary hormones for females and males, respectively (Morohashi et al., 2013). In our study, we demonstrated that letrozole caused an increase of estradiol serum levels in males in the primary hours after administration, and a long-term treatment did not present significant alterations in the estrogen and testosterone levels. Letrozole comprises a non-steroidal aromatase inhibitor, and its mechanism of action was supposed to decrease estradiol levels (Bhatnagar, 2007). The imbalance in the hormone levels in the pilot could be due to a compensatory mechanism that increases estradiol levels in the first hours. Letrozole is reported to be rapidly absorbed by the gut and has a half-life of about one day (Haynes et al., 2003). However, the long-term treatment did not affect the testosterone/estradiol ratio in the treated animals, which could be attributed to males already presenting lower levels of estradiol and higher levels of testosterone (Carreau et al., 2007).

The expression analysis revealed no differences in the aromatase gene expression in the testis of treated animals compared to the control group. The primary source of aromatase is Leydig cells (Zirkin and Papadopoulos, 2018), which the positive maker can be observed in immunohistochemistry analysis. The aromatase expression in germ cells has been reported in several studies with different mammals (Bourguiba et al., 2003; Conley and Hinshelwood, 2001). In addition, we also observed that aromatase was usually found surrounding the germ cell nucleus. Germ cells have high estrogen levels and probably present high expression levels of the aromatase gene (Carreau et al., 2012, 2011). Nevertheless, as letrozole acts as an aromatase inhibitor, its action may not affect the gene expression in somatic and germ cells from the testis, as we observed in our analysis.

Despite those effects, the letrozole also increased the testis circumference and the size of the seminiferous tubules in the treated animals. Letrozole has been used to treat male infertility for years, and it has different causes, such as autoimmune cases and steroid hormone deficiency (Schlegel, 2012). Also, male infertility has been demonstrated in mammalian models, including ovine (Lees, 1978; Santolaria et al., 2011). As ovine reproduction in the Northeast region of Brazil has been difficult in captivity, we hypothesized whether letrozole would have effects on ovine male fertility (Katiele et al., 2020; McManus et al., 2010; Shelton and Figueiredo, 1990). Although letrozole treatment had apparent effects on steroid hormones, there were no observable effects on the reproductive parameters analyzed in our study, such as the semen volume, sperm concentration, or spermatozoa characteristics.

On the other hand, side effects or tissue damage by letrozole treatment on spermatozoa were also expected. However, we did not detect any pathological signs in the testis or damage to the spermatozoa produced by the treated animals revealed by the histological analysis. Moreover, the imbalance of testosterone/estradiol levels by the long-term letrozole treatment can cause a reverse effect in treated animals. Prolongated letrozole use was reported to delay the development of seminiferous tubules and spermatogenesis (Maria Angelica Machado Arroyo et al., 2021). In addition, higher testosterone levels can completely sterilize the animal, as reported by several studies in mammals and fishes (Solar and Donaldson, 1985; Vanderstichel et al., 2015), which was not observed in our case.

## Conclusion

In summary, our results showed that letrozole oral treatment induces changes in the estradiol/testosterone ratio in the first hours of treatment. However, a long-term administration of these effects has yet to be observed. In addition, letrozole induces testicular size and seminiferous tubule growth. Nevertheless, there was no alteration in the reproductive parameters of treatment animals, such as sperm concentration or motility. Although the toxic effects of letrozole treatment were not observed in the testicular tissue or spermatozoa cells even after a long-term treatment. Letrozole comprises a non-steroid aromatase inhibitor, which has several uses and can also be applied to ovine reproduction. Exploring other administration methods, such as subcutaneous or intramuscular injections, could help to improve the use of letrozole in ovine models.
